# The Secret of Success

**Published:** 1879-08

**Authors:** 


THE SECRET OF SUCCESS depends on fair dealing, thorough knowledge of business, and strict attention to it. Some years ago, by mere chance, we sent some orders for goods to Messrs. Hall & Ruckel, No. 218 Greenwich Street, one of the oldest and largest wholesale drug houses in New York. Everything was so satisfactory that we have continued to deal with them since; and we can easily understand why they number their customers by thousands. This firm manufactures the "HOLLOW SUPPOSITORIES" advertised in this JOURNAL, and that fact is a guarantee that all orders for an article for which there is so large a demand will be promptly filled. The "HOLLOW SUPPOSITORIES" are neatly put up in boxes containing half a gross each. The price of a half gross box, size No. 1, is $1.50. Size No. 2, is $1.75. Size No. 3 is $2.00. Sent free by mail on receipt of price.

